# 4,4′-[(5-Carb­oxy-1,3-phenyl­ene)bis­(­oxy)]dibenzoic acid

**DOI:** 10.1107/S1600536812012275

**Published:** 2012-03-31

**Authors:** Chao Du, Wei Wu, Ge Tian

**Affiliations:** a Second Department of Neurosurgery, Bethune Third Hospital (China–Japan Union Hospital), Jilin University, People’s Republic of China; b Radiological Department, Tumor Hospital of Jilin Province, People’s Republic of China; c State Key Laboratory of Inorganic Synthesis and Preparative Chemistry, College of Chemistry, Jilin University, Changchun 130012, People’s Republic of China

## Abstract

In the title compound, C_21_H_14_O_8_, the central benzene ring makes dihedral angles of 77.8 (6) and 75.9 (5)° with the outer benzene rings. In the crystal, mol­ecules are linked by O—H⋯O hydrogen bonds involving carboxyl groups, forming one-dimensional ladders. Two-dimensional layers are formed by inter­penetration of these one-dimensional ladders.

## Related literature

For general background, see: Moulton & Zaworotko,(2001 ▶); Kitagawa *et al.*,(2001 ▶); Lee *et al.*,(2009 ▶); Robin & Fromm, (2006 ▶). For the preparation of title compound, see: Neogi *et al.*(2009 ▶). For related structa­res, see: Lama *et al.*(2010 ▶); Pan *et al.* (2007 ▶).
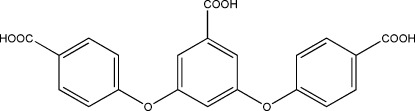


## Experimental

### 

#### Crystal data

C_21_H_14_O_8_
*M* *_r_* = 394.32
Monoclinic, 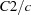

*a* = 17.235 (3) Å
*b* = 13.419 (3) Å
*c* = 15.586 (3) Å
β = 96.24 (3)°
*V* = 3583.3 (12) Å^3^
*Z* = 8
Mo *K*α radiation
μ = 0.11 mm^−1^
*T* = 293 K
0.33 × 0.29 × 0.25 mm

#### Data collection

Bruker SMART CCD area-detector diffractometer
Absorption correction: multi-scan (*SADABS*; Bruker, 2003 ▶) *T*_min_ = 0.316, *T*_max_ = 0.622
16922 measured reflections
4073 independent reflections
2862 reflections with *I* > 2σ(*I*)
*R*_int_ = 0.046

#### Refinement

*R*[*F* ^2^ > 2σ(*F* ^2^)] = 0.046
*wR*(*F* ^2^) = 0.121
*S* = 1.09
4073 reflections
262 parameters
H-atom parameters constrained
Δρ_max_ = 0.28 e Å^−3^
Δρ_min_ = −0.22 e Å^−3^

### 

Data collection: *SMART* (Bruker, 2001 ▶); cell refinement: *SAINT-Plus* (Bruker, 2003 ▶); data reduction: *SAINT-Plus*; program(s) used to solve structure: *SHELXS97* (Sheldrick, 2008 ▶); program(s) used to refine structure: *SHELXL97* (Sheldrick, 2008 ▶); molecular graphics: *SHELXP97* (Sheldrick, 2008 ▶); software used to prepare material for publication: *SHELXTL*.

## Supplementary Material

Crystal structure: contains datablock(s) global, I. DOI: 10.1107/S1600536812012275/bq2343sup1.cif

Structure factors: contains datablock(s) I. DOI: 10.1107/S1600536812012275/bq2343Isup2.hkl

Supplementary material file. DOI: 10.1107/S1600536812012275/bq2343Isup3.cml

Additional supplementary materials:  crystallographic information; 3D view; checkCIF report

## Figures and Tables

**Table 1:** Hydrogen-bond geometry (Å, °)

*D*—H⋯*A*	*D*—H	H⋯*A*	*D*⋯*A*	*D*—H⋯*A*
O3—H6⋯O4^i^	0.82	1.87	2.6919 (16)	176.4
O6—H3⋯O8^ii^	0.82	1.85	2.6615 (18)	169.9
O7—H8⋯O5^iii^	0.82	1.82	2.6307 (18)	167.2

Symmetry codes: (i) 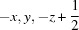; (ii) 
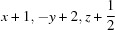
; (iii) 
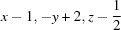
.

**Fig. 1.**
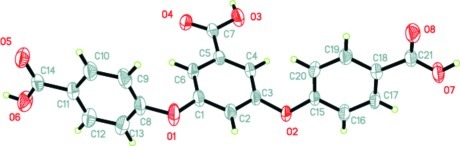
The molecular structure of the title compound with the atom-numbering scheme, with 50% probability displacement ellipsoids.

**Fig. 2.**
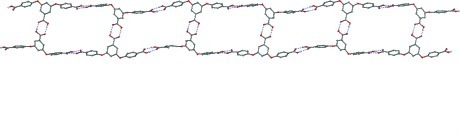
The packing of title compound, showing one ladder of molecules connected by O—H···O hydrogen bonds (dashed lines). H atoms not involved in hydrogen bonding have been omitted for clarity.

**Fig. 3.**
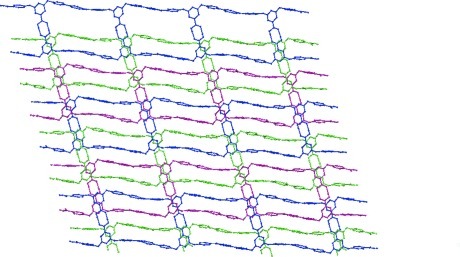
The interpenetration among the one-dimensional ladders, showing two-dimensional layer. H atoms not involved in hydrogen bonding have been omitted for clarity.

## supplementary crystallographic information

### Comment

As a new kind of functional molecular materials, metal-organic frameworks have received extensive attention for their potential applications in gas storage, catalysis, optoelectronics, sensors, magnetism, luminescence, porous materials and so on. (Moulton & Zaworotko, 2001; Kitagawa *et al.*, 2001; Lee *et al.*, 2009). Organic molecules with O– and N-donors can be used as organic linkers in these coordination polymers (Robin & Fromm, 2006). In fact, there are many organic ligands which are linked by ether bond (Lama *et al.* 2010; Pan *et al.*, 2007). Here, we report the crystal structure of the title compound.

In the crystal structure, two benzene rings, β(composed of C_8_—C_13_) and γ (composed of C_15_—C_20_) are connected to the center ring (α, composed of C_1_—C_6_) by ether bond. The dihedral angle between α and β is 77.8 (6)°, and between α and γ is 75.9 (5)° (Fig. 1). Strong intermolecular O—H···O hydrogen bonds are formed between the carboxylic acid groups of neighboring molecules (Table 1), which link the molecules to one-dimensional supra-molecular ladder (Fig. 2). The interpenetration among the one-dimensional molecular ladders which are parallel produce two-dimensional layer (Fig. 3).

### Experimental

The title compound was synthesized by a modified literature method (Neogi *et al.*2009). Methyl 3,5-dihydroxylbenzoate (1.68 g,10 mmol) was dissolved in DMF (50 ml). To this solution was added K_2_CO_3_ (7 g,51 mmol) and 4-fluorobenzonitrile (2.4 g,20 mmol). The mixture was heated under reflux for 2 days. The resulting solution was poured in 250 ml ice-cold water and kept over-night. The yellow compound was filtered and washed several times with water. The yellow compound (3.73 g, 10 mmol) was allowed to reflux with 6 N NaOH solution (50 ml) for 12 h, cooled to room temperature and acidified with HCl (6 N). Colorless crystalline product was obtained and isolated by filtration, washed with water and dried in vacuum. Zn(NO_3_)_2_ (0.075 g,0.25 mmol), 4,4'-(5-carboxy-1,3-phenylene)bis(oxy)dibenzoic acid (0.098 g,0.25 mmol), were mixed in water (5 ml). The mixture were placed in a 25 ml Teflon-lined stainless steel autoclave and heated autogenously under pressure for 2 d at 393 K. After cooling to room temperature, the block-shaped colourless crystals were obtained.

### Refinement

All hydrogen atoms bonded to O and C were fixed in ideal positions, with C—H = 0.93 (aromatic) and O—H = 0.82 Å, and treated as riding on their parent atoms with *U*_iso_(H)=0.08 Å^2^.

### Crystal data

**Table:**

C_21_H_14_O_8_	*F*(000) = 1632
*M**_r_* = 394.32	*D*_x_ = 1.462 Mg m^−^^3^
Monoclinic, *C*2/*c*	Mo *K*α radiation, λ = 0.71073 Å
Hall symbol: -C 2yc	Cell parameters from 4073 reflections
*a* = 17.235 (3) Å	θ = 3.0–27.5°
*b* = 13.419 (3) Å	µ = 0.11 mm^−^^1^
*c* = 15.586 (3) Å	*T* = 293 K
β = 96.24 (3)°	Block, colourless
*V* = 3583.3 (12) Å^3^	0.33 × 0.29 × 0.25 mm
*Z* = 8	

### Data collection

**Table:**

Bruker SMART CCD area-detector diffractometer	4073 independent reflections
Radiation source: fine-focus sealed tube	2862 reflections with *I* > 2σ(*I*)
Graphite monochromator	*R*_int_ = 0.046
phi and ω scans	θ_max_ = 27.5°, θ_min_ = 3.0°
Absorption correction: multi-scan (*SADABS*; Bruker, 2003)	*h* = −22→22
*T*_min_ = 0.316, *T*_max_ = 0.622	*k* = −17→17
16922 measured reflections	*l* = −19→20

### Refinement

**Table:**

Refinement on *F*^2^	Primary atom site location: structure-invariant direct methods
Least-squares matrix: full	Secondary atom site location: difference Fourier map
*R*[*F*^2^ > 2σ(*F*^2^)] = 0.046	Hydrogen site location: inferred from neighbouring sites
*wR*(*F*^2^) = 0.121	H-atom parameters constrained
*S* = 1.09	*w* = 1/[σ^2^(*F*_o_^2^) + (0.0601*P*)^2^ + 0.3436*P*] where *P* = (*F*_o_^2^ + 2*F*_c_^2^)/3
4073 reflections	(Δ/σ)_max_ < 0.001
262 parameters	Δρ_max_ = 0.28 e Å^−^^3^
0 restraints	Δρ_min_ = −0.22 e Å^−^^3^

### Special details

**Table:**

Geometry. All e.s.d.'s (except the e.s.d. in the dihedral angle between two l.s. planes) are estimated using the full covariance matrix. The cell e.s.d.'s are taken into account individually in the estimation of e.s.d.'s in distances, angles and torsion angles; correlations between e.s.d.'s in cell parameters are only used when they are defined by crystal symmetry. An approximate (isotropic) treatment of cell e.s.d.'s is used for estimating e.s.d.'s involving l.s. planes.
Refinement. Refinement of *F*^2^ against ALL reflections. The weighted *R*-factor *wR* and goodness of fit *S* are based on *F*^2^, conventional *R*-factors *R* are based on *F*, with *F* set to zero for negative *F*^2^. The threshold expression of *F*^2^ > σ(*F*^2^) is used only for calculating *R*-factors(gt) *etc*. and is not relevant to the choice of reflections for refinement. *R*-factors based on *F*^2^ are statistically about twice as large as those based on *F*, and *R*- factors based on ALL data will be even larger.

### Fractional atomic coordinates and isotropic or equivalent isotropic displacement parameters (Å^2^)

**Table:**

	*x*	*y*	*z*	*U*_iso_*/*U*_eq_	
O1	0.16748 (7)	0.89288 (12)	−0.11510 (7)	0.0630 (4)	
O2	−0.07879 (6)	0.73896 (9)	−0.14922 (7)	0.0404 (3)	
O3	−0.06065 (6)	0.88133 (11)	0.15033 (7)	0.0503 (4)	
H6	−0.0624	0.8964	0.2011	0.080*	
O4	0.06498 (6)	0.92236 (9)	0.18120 (7)	0.0386 (3)	
O5	0.44526 (7)	1.12170 (10)	0.10342 (8)	0.0533 (3)	
O6	0.48890 (7)	0.96517 (11)	0.10924 (10)	0.0622 (4)	
H3	0.5260	0.9916	0.1379	0.080*	
O7	−0.42509 (6)	0.81151 (10)	−0.31119 (8)	0.0517 (3)	
H8	−0.4653	0.8389	−0.3320	0.080*	
O8	−0.38899 (7)	0.96958 (10)	−0.28672 (8)	0.0510 (3)	
C1	0.10101 (8)	0.87058 (14)	−0.07678 (10)	0.0383 (4)	
C2	0.04339 (9)	0.82152 (13)	−0.12950 (10)	0.0389 (4)	
H11A	0.0509	0.8057	−0.1861	0.080*	
C3	−0.02518 (8)	0.79647 (12)	−0.09712 (10)	0.0335 (4)	
C4	−0.03815 (8)	0.82052 (12)	−0.01343 (10)	0.0338 (4)	
H9A	−0.0852	0.8047	0.0074	0.080*	
C5	0.02053 (8)	0.86866 (12)	0.03845 (9)	0.0305 (3)	
C6	0.09117 (8)	0.89385 (12)	0.00791 (9)	0.0340 (4)	
H13A	0.1305	0.9254	0.0435	0.080*	
C7	0.01100 (8)	0.89365 (12)	0.13006 (10)	0.0318 (3)	
C8	0.23310 (9)	0.92824 (16)	−0.06384 (10)	0.0457 (5)	
C9	0.24061 (9)	1.02836 (17)	−0.04567 (12)	0.0520 (5)	
H3A	0.2011	1.0727	−0.0652	0.080*	
C10	0.30817 (10)	1.06221 (15)	0.00240 (12)	0.0475 (4)	
H2A	0.3139	1.1296	0.0154	0.080*	
C11	0.36696 (8)	0.99590 (14)	0.03100 (10)	0.0379 (4)	
C12	0.35866 (10)	0.89573 (15)	0.01005 (12)	0.0491 (5)	
H6A	0.3984	0.8512	0.0282	0.080*	
C13	0.29151 (10)	0.86173 (16)	−0.03783 (11)	0.0510 (5)	
H5A	0.2860	0.7946	−0.0522	0.080*	
C14	0.43795 (9)	1.03153 (14)	0.08419 (11)	0.0393 (4)	
C15	−0.15218 (8)	0.77687 (12)	−0.17470 (9)	0.0310 (3)	
C16	−0.20659 (9)	0.70800 (13)	−0.21092 (11)	0.0396 (4)	
H17A	−0.1938	0.6408	−0.2131	0.080*	
C17	−0.27973 (9)	0.74089 (13)	−0.24351 (11)	0.0407 (4)	
H16A	−0.3166	0.6952	−0.2673	0.080*	
C18	−0.29914 (8)	0.84138 (13)	−0.24126 (10)	0.0340 (4)	
C19	−0.24389 (9)	0.90878 (13)	−0.20428 (10)	0.0380 (4)	
H20A	−0.2565	0.9761	−0.2023	0.080*	
C20	−0.17048 (9)	0.87698 (13)	−0.17049 (10)	0.0367 (4)	
H19A	−0.1340	0.9223	−0.1453	0.080*	
C21	−0.37538 (9)	0.87785 (13)	−0.28147 (10)	0.0376 (4)	

### Atomic displacement parameters (Å^2^)

**Table:**

	*U*^11^	*U*^22^	*U*^33^	*U*^12^	*U*^13^	*U*^23^
O1	0.0313 (6)	0.1242 (13)	0.0339 (6)	−0.0307 (7)	0.0050 (5)	−0.0151 (7)
O2	0.0226 (5)	0.0450 (7)	0.0501 (7)	0.0008 (5)	−0.0117 (5)	−0.0106 (5)
O3	0.0275 (6)	0.0879 (10)	0.0359 (6)	−0.0055 (6)	0.0051 (5)	−0.0026 (6)
O4	0.0332 (6)	0.0474 (7)	0.0347 (6)	−0.0087 (5)	0.0009 (5)	−0.0024 (5)
O5	0.0394 (7)	0.0487 (9)	0.0676 (9)	−0.0075 (6)	−0.0131 (6)	−0.0023 (6)
O6	0.0382 (7)	0.0610 (10)	0.0805 (9)	0.0024 (7)	−0.0247 (6)	−0.0065 (7)
O7	0.0291 (6)	0.0479 (8)	0.0730 (9)	0.0008 (5)	−0.0179 (6)	−0.0011 (6)
O8	0.0401 (7)	0.0423 (8)	0.0657 (8)	0.0062 (6)	−0.0167 (6)	−0.0016 (6)
C1	0.0226 (7)	0.0587 (12)	0.0329 (8)	−0.0054 (7)	0.0002 (6)	0.0002 (8)
C2	0.0266 (8)	0.0567 (12)	0.0317 (8)	−0.0007 (7)	−0.0045 (6)	−0.0038 (7)
C3	0.0207 (7)	0.0385 (9)	0.0386 (8)	0.0009 (6)	−0.0088 (6)	−0.0009 (7)
C4	0.0215 (7)	0.0412 (10)	0.0378 (8)	−0.0012 (6)	−0.0016 (6)	0.0027 (7)
C5	0.0242 (7)	0.0348 (9)	0.0316 (7)	0.0010 (6)	−0.0014 (6)	0.0048 (6)
C6	0.0242 (7)	0.0442 (10)	0.0320 (8)	−0.0050 (7)	−0.0036 (6)	0.0010 (7)
C7	0.0254 (7)	0.0353 (9)	0.0340 (8)	0.0012 (6)	0.0000 (6)	0.0043 (6)
C8	0.0257 (8)	0.0812 (15)	0.0303 (8)	−0.0177 (9)	0.0038 (7)	−0.0046 (9)
C9	0.0272 (8)	0.0777 (15)	0.0488 (10)	0.0032 (9)	−0.0062 (7)	0.0007 (10)
C10	0.0311 (9)	0.0559 (12)	0.0537 (11)	−0.0020 (8)	−0.0039 (8)	−0.0007 (9)
C11	0.0259 (8)	0.0483 (11)	0.0383 (8)	−0.0064 (7)	−0.0018 (6)	0.0044 (8)
C12	0.0402 (9)	0.0508 (12)	0.0537 (11)	−0.0071 (8)	−0.0072 (8)	0.0019 (9)
C13	0.0452 (10)	0.0584 (13)	0.0481 (10)	−0.0179 (9)	−0.0011 (9)	−0.0028 (9)
C14	0.0264 (8)	0.0454 (11)	0.0444 (9)	−0.0010 (7)	−0.0031 (7)	0.0028 (8)
C15	0.0211 (7)	0.0401 (9)	0.0307 (7)	−0.0023 (6)	−0.0026 (6)	0.0010 (7)
C16	0.0287 (8)	0.0352 (10)	0.0518 (10)	−0.0024 (7)	−0.0098 (7)	−0.0023 (7)
C17	0.0260 (8)	0.0401 (10)	0.0526 (10)	−0.0063 (7)	−0.0106 (7)	−0.0001 (8)
C18	0.0246 (7)	0.0408 (10)	0.0353 (8)	−0.0009 (7)	−0.0033 (6)	0.0021 (7)
C19	0.0325 (8)	0.0379 (10)	0.0412 (9)	0.0015 (7)	−0.0065 (7)	0.0004 (7)
C20	0.0289 (8)	0.0396 (10)	0.0394 (8)	−0.0070 (7)	−0.0065 (7)	−0.0021 (7)
C21	0.0265 (8)	0.0438 (11)	0.0406 (9)	−0.0007 (7)	−0.0043 (7)	−0.0004 (7)

### Geometric parameters (Å, º)

**Table:**

O1—C1	1.3811 (18)	C8—C13	1.373 (3)
O1—C8	1.395 (2)	C8—C9	1.376 (3)
O2—C15	1.3810 (17)	C9—C10	1.391 (2)
O2—C3	1.3949 (18)	C9—H3A	0.9300
O3—C7	1.3179 (17)	C10—C11	1.385 (2)
O3—H6	0.8200	C10—H2A	0.9300
O4—C7	1.2199 (19)	C11—C12	1.387 (3)
O5—C14	1.250 (2)	C11—C14	1.481 (2)
O6—C14	1.281 (2)	C12—C13	1.384 (2)
O6—H3	0.8200	C12—H6A	0.9300
O7—C21	1.286 (2)	C13—H5A	0.9300
O7—H8	0.8200	C15—C20	1.383 (2)
O8—C21	1.254 (2)	C15—C16	1.392 (2)
C1—C6	1.385 (2)	C16—C17	1.379 (2)
C1—C2	1.385 (2)	C16—H17A	0.9300
C2—C3	1.376 (2)	C17—C18	1.391 (2)
C2—H11A	0.9300	C17—H16A	0.9300
C3—C4	1.385 (2)	C18—C19	1.392 (2)
C4—C5	1.384 (2)	C18—C21	1.476 (2)
C4—H9A	0.9300	C19—C20	1.384 (2)
C5—C6	1.396 (2)	C19—H20A	0.9300
C5—C7	1.493 (2)	C20—H19A	0.9300
C6—H13A	0.9300		
			
C1—O1—C8	119.01 (12)	C9—C10—H2A	119.9
C15—O2—C3	119.45 (12)	C10—C11—C12	119.59 (16)
C7—O3—H6	109.5	C10—C11—C14	120.24 (17)
C14—O6—H3	109.5	C12—C11—C14	120.17 (15)
C21—O7—H8	109.5	C13—C12—C11	120.33 (18)
O1—C1—C6	123.82 (14)	C13—C12—H6A	119.8
O1—C1—C2	114.92 (13)	C11—C12—H6A	119.8
C6—C1—C2	121.25 (14)	C8—C13—C12	119.21 (19)
C3—C2—C1	119.18 (14)	C8—C13—H5A	120.4
C3—C2—H11A	120.4	C12—C13—H5A	120.4
C1—C2—H11A	120.4	O5—C14—O6	123.49 (16)
C2—C3—C4	121.42 (14)	O5—C14—C11	120.18 (15)
C2—C3—O2	117.55 (13)	O6—C14—C11	116.32 (16)
C4—C3—O2	120.84 (13)	O2—C15—C20	123.41 (14)
C5—C4—C3	118.45 (13)	O2—C15—C16	115.32 (14)
C5—C4—H9A	120.8	C20—C15—C16	121.14 (14)
C3—C4—H9A	120.8	C17—C16—C15	119.03 (16)
C4—C5—C6	121.50 (14)	C17—C16—H17A	120.5
C4—C5—C7	120.94 (13)	C15—C16—H17A	120.5
C6—C5—C7	117.54 (14)	C16—C17—C18	120.92 (15)
C1—C6—C5	118.17 (14)	C16—C17—H16A	119.5
C1—C6—H13A	120.9	C18—C17—H16A	119.5
C5—C6—H13A	120.9	C17—C18—C19	119.00 (14)
O4—C7—O3	123.42 (14)	C17—C18—C21	121.08 (14)
O4—C7—C5	122.71 (13)	C19—C18—C21	119.84 (15)
O3—C7—C5	113.86 (13)	C20—C19—C18	120.88 (16)
C13—C8—C9	121.61 (16)	C20—C19—H20A	119.6
C13—C8—O1	118.08 (19)	C18—C19—H20A	119.6
C9—C8—O1	120.15 (18)	C15—C20—C19	119.02 (15)
C8—C9—C10	118.97 (17)	C15—C20—H19A	120.5
C8—C9—H3A	120.5	C19—C20—H19A	120.5
C10—C9—H3A	120.5	O8—C21—O7	122.91 (15)
C11—C10—C9	120.25 (19)	O8—C21—C18	120.30 (14)
C11—C10—H2A	119.9	O7—C21—C18	116.78 (15)

### Hydrogen-bond geometry (Å, º)

**Table:**

*D*—H···*A*	*D*—H	H···*A*	*D*···*A*	*D*—H···*A*
O3—H6···O4^i^	0.82	1.87	2.6919 (16)	176.4
O6—H3···O8^ii^	0.82	1.85	2.6615 (18)	169.9
O7—H8···O5^iii^	0.82	1.82	2.6307 (18)	167.2

Symmetry codes: (i) −*x*, *y*, −*z*+1/2; (ii) *x*+1, −*y*+2, *z*+1/2; (iii) *x*−1, −*y*+2, *z*−1/2.
